# Predicting COVID-19 Pneumonia Severity on Chest X-ray With Deep Learning

**DOI:** 10.7759/cureus.9448

**Published:** 2020-07-28

**Authors:** Joseph Paul Cohen, Lan Dao, Karsten Roth, Paul Morrison, Yoshua Bengio, Almas F Abbasi, Beiyi Shen, Hoshmand Kochi Mahsa, Marzyeh Ghassemi, Haifang Li, Tim Q Duong

**Affiliations:** 1 Department of Computer Science, University of Montreal, Montreal, CAN; 2 Medicine, University of Montreal, Montreal, CAN; 3 Department of Computer Science, Heidelberg University, Heidelberg, DEU; 4 Department of Mathematics and Computer Science, Fontbonne University, Saint Louis, USA; 5 Department of Radiology, Stony Brook Medicine, Stony Brook, USA; 6 Department of Computer Science, University of Toronto, Toronto, CAN

**Keywords:** chest x-ray, covid-19 pneumonia, severity scoring, deep learning artificial intelligence

## Abstract

Introduction

The need to streamline patient management for coronavirus disease-19 (COVID-19) has become more pressing than ever. Chest X-rays (CXRs) provide a non-invasive (potentially bedside) tool to monitor the progression of the disease. In this study, we present a severity score prediction model for COVID-19 pneumonia for frontal chest X-ray images. Such a tool can gauge the severity of COVID-19 lung infections (and pneumonia in general) that can be used for escalation or de-escalation of care as well as monitoring treatment efficacy, especially in the ICU.

Methods

Images from a public COVID-19 database were scored retrospectively by three blinded experts in terms of the extent of lung involvement as well as the degree of opacity. A neural network model that was pre-trained on large (non-COVID-19) chest X-ray datasets is used to construct features for COVID-19 images which are predictive for our task.

Results

This study finds that training a regression model on a subset of the outputs from this pre-trained chest X-ray model predicts our geographic extent score (range 0-8) with 1.14 mean absolute error (MAE) and our lung opacity score (range 0-6) with 0.78 MAE.

Conclusions

These results indicate that our model’s ability to gauge the severity of COVID-19 lung infections could be used for escalation or de-escalation of care as well as monitoring treatment efficacy, especially in the ICU. To enable follow up work, we make our code, labels, and data available online.

## Introduction

As the first countries explore deconfinement strategies [1], the death toll of coronavirus disease-19 (COVID-19) keeps rising [2]. The increased strain caused by the pandemic on healthcare systems worldwide has prompted many physicians to resort to new strategies and technologies. Chest X-rays (CXRs) provide a non-invasive (potentially bedside) tool to monitor the progression of the disease [3,4]. As early as March 2020, Chinese hospitals used artificial intelligence (AI)-assisted computed tomography (CT) imaging analysis to screen COVID-19 cases and streamline diagnosis [5]. Many teams have since launched AI initiatives to improve triaging of COVID-19 patients (i.e., discharge, general admission, or ICU care) and allocation of hospital resources (i.e., direct non-invasive ventilation to invasive ventilation) [6]. While these recent tools exploit clinical data, practically deployable CXR-based predictive models remain lacking. 

In this work, we built and studied a model which predicts the severity of COVID-19 pneumonia, based on CXRs, to be used as an assistive tool when managing patient care. The ability to gauge the severity of COVID-19 lung infections can be used for escalation or de-escalation of care, especially in the ICU. An automated tool can be applied to patients over time to objectively and quantitatively track disease progression and treatment response.

## Materials and methods

2.1 COVID-19 cohort 

We used a retrospective cohort of 94 posteroanterior (PA) CXR images from a public COVID-19 image data collection [7]. While the dataset currently contains 153 images, it only counted 94 images at the time of the experiment, all of which were included in the study. All patients were reported as COVID-19 positive by their physicians (most using RT-PCR) and sourced from many hospitals around the world from December 2019 to March 2020. The images were de-identified prior to our use and there was no missing data. The ratio between male/female was 44/36 with an average age of 56±14.8 (55±15.6 for male and 57±13.9 for female). 

2.2 Labels 

Radiological scoring was performed by three blinded experts: two chest radiologists (each with at least 20 years of experience) and a radiology resident. They staged disease severity using a score system [8], based on two types of scores (parameters): extent of lung involvement and degree of opacity. They were only presented with a single CXR image at a time without any clinical context of the patient.

1. The extent of lung involvement by ground glass opacity or consolidation for each lung (right lung and left lung separately) was scored as: 0 = no involvement; 1 = <25% involvement; 2 = 25%-50% involvement; 3 = 50%-75% involvement; 4 = >75% involvement. The total extent score ranged from 0 to 8 (right lung and left lung together). 

2. The degree of opacity for each lung (right lung and left lung separately) was scored as: 0 = no opacity; 1 = ground glass opacity; 2 = consolidation; 3 = white-out. The total opacity score ranged from 0 to 6 (right lung and left lung together). 

A spreadsheet was maintained to pair filenames with their respective scores. Fleiss’ Kappa for inter-rater agreement was 0.45 for the opacity score and 0.71 for the extent score.

2.3 Non-COVID-19 (pre-training) datasets 

Prior to the experiment, the model was trained on the following public datasets, none of which contained COVID-19 cases: 

- Radiological Society of North America (RSNA) Pneumonia Detection Challenge [9]; - CheXpert dataset from Stanford University [10]; - ChestX-ray8 dataset from the National Institute of Health (NIH) [11]; - ChestX-ray8 dataset from the NIH with labels from Google [12]; - MIMIC-CXR dataset from Massachusetts Institute of Technology (MIT) [13]; - PadChest dataset from the University of Alicante [14]; - OpenI [15] 

These seven datasets were manually aligned to each other on 18 common radiological finding tasks in order to train a model using all datasets at once (atelectasis, consolidation, infiltration, pneumothorax, edema, emphysema, fibrosis, fibrosis, effusion, pneumonia, pleural thickening, cardiomegaly, nodule, mass, hernia, lung lesion, fracture, lung opacity, and enlarged cardiomediastinum). For example “pleural effusion” from one dataset is considered the same as “effusion” from another dataset in order to consider these labels equal. In total, 88,079 non-COVID-19 images were used to train the model on these tasks. 

2.4 Model, preprocessing, and pre-training 

In this study, we used a DenseNet model [16] from the TorchXRayVision library [17,18]. DenseNet models have been shown to predict pneumonia well [19]. Images were resized to 224x224 pixels, utilizing a center crop if the aspect ratio was uneven, and the pixel values were scaled to (-1024, 1024) for the training. 

**Figure 1:**
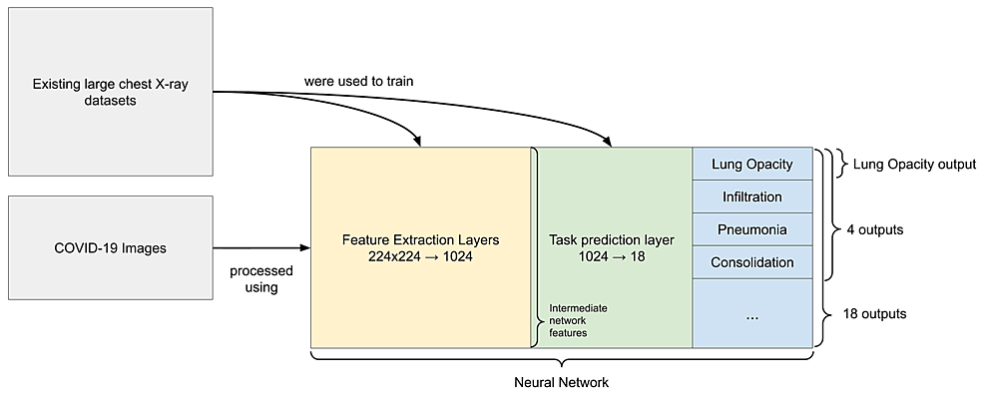
Diagram of features extracted from the images The two dataset blocks show that COVID-19 images were not used to train the neural network. The network diagram is split into three sections. The feature extraction layers are convolutional layers which transform the image into a 1024 dimensional vector which is called the intermediate network features. These features are then transformed using the task prediction layer (a sigmoid function for each task) into the outputs for each task. The different groupings of outputs used in this work are shown.

Before even processing the COVID-19 images, a pre-training step was performed using the seven datasets to train feature extraction layers and a task prediction layer (Figure 1). This “pre-training” step was performed on a large set of data in order to construct general representations about lungs and other aspects of CXRs that we would have been unable to achieve on the small set of COVID-19 images available. Some of these representations are expected to be relevant to our downstream tasks. There are a few ways we can extract useful features from the pre-trained model as detailed in Figure 1. 

2.5 Training 

Similarly to the images from non-COVID-19 datasets used for pre-training, each image from the COVID-19 dataset was preprocessed (resized, center cropped, rescaled), then processed by the feature extraction layers and the task prediction layer of the network. The network was trained on existing datasets before the weights were frozen. COVID-19 images were processed by the network to generate features used in place of the images. As was the case with images from the seven non-COVID-19 datasets, the feature extraction layers produced a representation of the 94 COVID-19 images using a 1024 dimensional vector, then the fully connected task prediction layer produced outputs for each of the 18 original tasks. We build models on the pre-sigmoid outputs. 

Linear regression was performed to predict the aforementioned scores (extent of lung involvement and opacity) using these different sets of features in place of the image itself: 

- Intermediate network features - the result of the convolutional layers applied to the image resulting in a 1024 dimensional vector which is passed to the task prediction layer; - 18 outputs - each image was represented by the 18 outputs (pre-sigmoid) from the pre-trained model; - Four outputs - a hand picked subset of outputs (pre-sigmoid) were used containing radiological findings more frequent in pneumonia (lung opacity, pneumonia, infiltration, and consolidation); - Lung opacity output - the single output (pre-sigmoid) for lung opacity was used because it was very related to this task. This feature was different from the opacity score that we would like to predict. 

For each experiment performed, the 94 images COVID-19 dataset was randomly split into a train and test set roughly 50/50. Multiple timepoints from the same patient were grouped together into the same split so that a patient did not span both sets. Sampling was repeated throughout training in order to obtain a mean and standard deviation for each performance. As linear regression was used, there was no early stopping that had to be done to prevent the model from overfitting. Therefore, the criterion for determining the final model was only the mean squared error (MSE) on the training set.

2.6 Saliency maps 

In order to ensure that the models are looking at reasonable aspects of the images [20-22], a saliency map is computed by computing the gradient of the output prediction with respect to the input image (if a pixel is changed, how much will it change the prediction). In order to smooth out the saliency map, it is blurred using a 5x5 Gaussian kernel. Keep in mind that these saliency maps have limitations and only offer a restricted view into why a model made a prediction [22,23].

## Results

3.1 Quantitative performance metrics 

The single “lung opacity” output as a feature yielded the best correlation (0.80), followed by four outputs (lung opacity, pneumonia, infiltration, and consolidation) parameters (0.79) (Table 1). Building a model on only a few outputs provides the best performance. The mean absolute error (MAE) is useful to understand the error in units of the scores that are predicted while the MSE helps to rank the different methods based on their furthest outliers. One possible reason that fewer features work best is that having fewer parameters prevents overfitting. Some features could serve as proxy variables to confounding attributes such as sex or age and preventing these features from being used prevents the distraction from hurting generalization performance. Hand selecting feature subsets which are intuitively related to this task imparts domain knowledge as a bias on the model which improves performance. Thus, the top performing model (using the single “lung opacity” output as a feature) is used for the subsequent qualitative analysis. 

**Table 1:** Performance metrics of each set of features for the Opacity Score and Geographic Extent prediction Evaluation is performed on 50 randomly chosen train test splits and the metrics here are computed on a hold out test set. R2: coefficient of determination; MAE: mean absolute error; MSE: mean squared error. “four outputs” refers to lung opacity, pneumonia, infiltration, and consolidation

Task	Using features:	# parameters (fewer is better)	Pearson Correlation	R^2^	MAE	MSE
Opacity Score	"lung opacity" output	1+1	0.78±0.04	0.58±0.09	0.78±0.05	0.86±0.11
	4 outputs	4+1	0.78±0.04	0.58±0.09	0.76±0.05	0.87±0.12
	18 outputs	18+1	0.73±0.09	0.44±0.16	0.86±0.11	1.15±0.33
	Intermediate network features	1024+1	0.66±0.08	0.25±0.21	1.01±0.09	1.54±0.28
	No data	0+1	-0.00±0.00	-0.08±0.10	1.24±0.10	2.26±0.36
Geographic Extent	"lung opacity" output	1+1	0.80±0.05	0.60±0.09	1.14±0.11	2.06±0.34
	4 outputs	4+1	0.79±0.05	0.57±0.10	1.19±0.11	2.17±0.37
	18 outputs	18+1	0.76±0.08	0.47±0.16	1.32±0.17	2.73±0.89
	Intermediate network features	1024+1	0.74±0.08	0.43±0.16	1.36±0.13	2.88±0.58
	No data	0+1	0.00±0.00	-0.08±0.10	2.00±0.17	5.60±0.95

3.2 Qualitative analysis of predicted scores 

**Figure 2:**
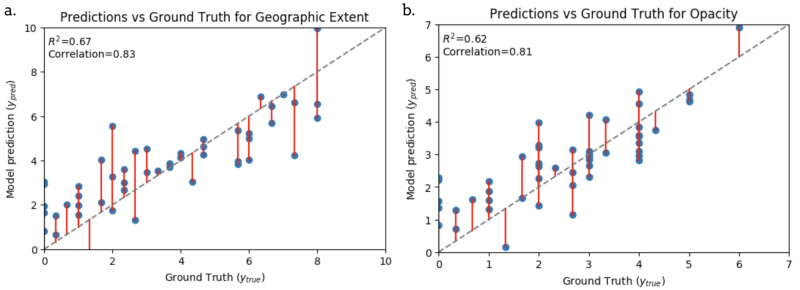
Scatter plots showing alignment between our best model predictions and human annotation (ground truth) for Geographical Extent and Opacity scores Evaluation is on a hold out test set. The grey dashed line is a perfect prediction. Red lines indicate error from a perfect prediction. R^2^: coefficient of determination.

Figure 2 shows the top performing model’s (using the single “lung opacity” output as a feature) predictions against the ground truth score (given by the blinded experts) on held out test data. Majority of the data points fall close to the line of unity. The model overestimates scores between 1 and 3 and underestimates scores above 4. However, generally the predictions seem reasonable given the agreement of the raters. 

3.3 Studying learned representations 

**Figure 3:**
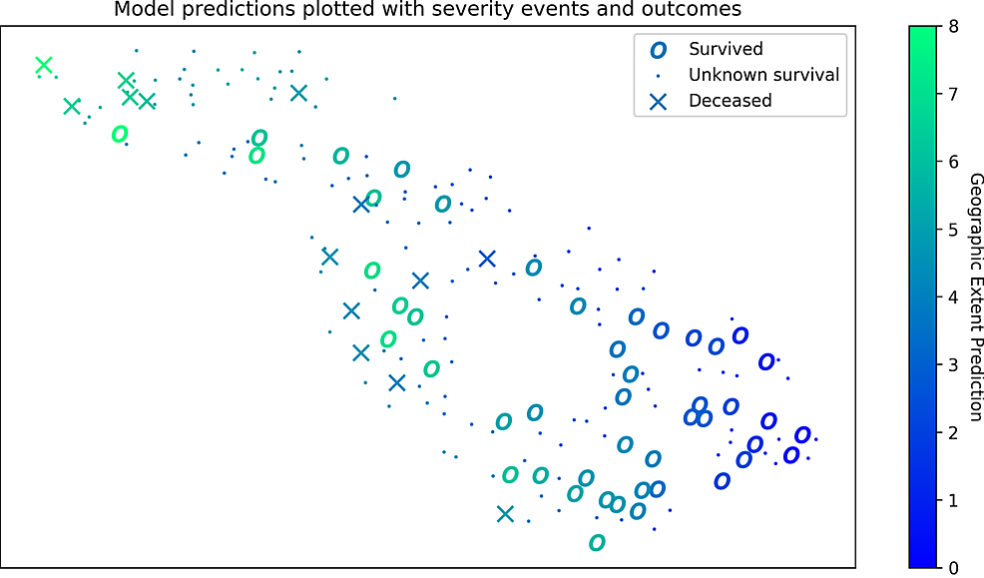
Feature representations visualized with geographic extent prediction and survival outcomes A spatial representation of pneumonia specific features (lung opacity, pneumonia, infiltration, and consolidation) when projected into 2 dimensions (2D) using a t-distributed stochastic neighbor embedding (t-SNE). In this 2D space, the high dimensional (4D) distances are preserved, specifically what is nearby. CXR images which have similar outputs are close to each other. Features are extracted for all 208 images in the dataset and the geographic extent prediction is shown for each image. The survival information available in the dataset represented by the shape of the marker.

In Figure 3, we explore what the representation used by one of the best models looks at in order to identify signs of overfitting and to gain insights into the variation of the data. A t-distributed stochastic neighbor embedding (t-SNE) [24] is computed on all data (even those not scored) in order to project the features into a two-dimensional (2D) space. Each CXR is represented by a point in a space where relationships to other points are preserved from the higher dimensional space. The cases of the survival group tend to cluster together as well as the cases of the deceased group. This clustering indicates that score predictions align with clinical outcomes. 

3.4 Inspecting saliency maps 

**Figure 4:**
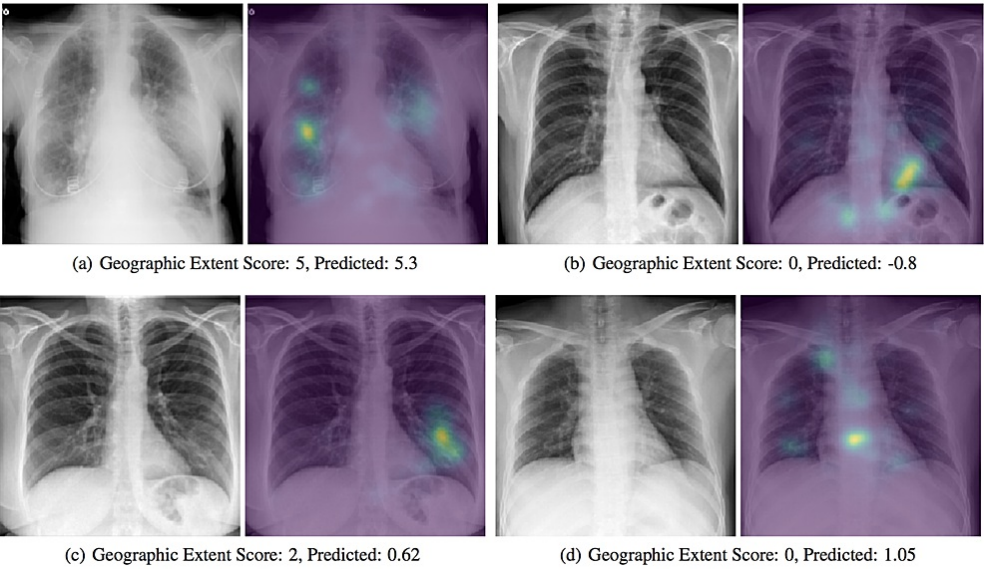
Saliency maps of model predictions Examples of correct (a,b) and incorrect (c,d) predictions by the model are shown with a saliency map generated by computing the gradient of the output prediction with respect to the input image and then blurred using a 5x5 Gaussian kernel. The assigned and predicted scores for Geographic Extent are shown to the right.

In Figure 4, images are studied which were not seen by the model during training. For most of the results, the model is correctly looking at opaque regions of the lungs. Figure 4b shows no signs of opacity and the model is focused on the heart and diaphragm, which is likely a sign that they are used as a color reference when determining what qualifies as opaque. In Figure 4c and Figure 4d, we see erroneous predictions.

## Discussion

In the context of a pandemic and the urgency to contain the crisis, research has increased exponentially in order to alleviate the healthcare system’s burden. However, many prediction models for diagnosis and prognosis of COVID-19 infection are at high risk of bias and model overfitting as well as poorly reported, their alleged performance being likely optimistic [25]. In order to prevent premature implementation in hospitals [26], tools must be robustly evaluated along several practical axes [18,27]. Indeed, while some AI-assisted tools might be powerful, they do not replace clinical judgment and their diagnostic performance cannot be assessed or claimed without a proper clinical trial [28]. 

Existing work focuses on predicting severity from a variety of clinical indicators which include findings from chest imaging [29]. Models such as the one presented in this work can complement and improve triage decisions from CXR as opposed to CT [30]. 

Challenges in creating a predictive model involve labelling the data and achieving good inter-rater agreement as well as learning a representation which will generalize to new images when the number of labelled images is so low. In the case of building a predictive tool for COVID-19 CXR images, the lack of a public database made it difficult to conduct large-scale robust evaluations. This small number of samples prevents proper cohort selection which is a limitation of this study and exposes our evaluation to sample bias. However, we use a model which was trained on a large dataset with related tasks which provided us with a robust unbiased COVID-19 feature extractor and allows us to learn only two parameters for our best linear regression model. Restricting the complexity of the learned model in this way reduces the possibility of overfitting. 

Our evaluation could be improved if we were able to obtain new cohorts labelled with the same severity score to ascertain the generalization of our model. Also, it is unknown if these radiographic scores of disease severity reflect actual functional or clinical outcomes as the open data do not have those data. We make the images, labels, model, and code public from this work so that other groups can perform follow-up evaluations.

## Conclusions

Our model’s ability to gauge the severity of COVID-19 lung infections could be used for escalation or de-escalation of care as well as monitoring treatment efficacy, especially in the ICU. The use of a score combining geographical extent and degree of opacity allows clinicians to compare CXR images with each other using a quantitative and objective measure. Also, this can be done at scale for a large scale analysis.
